# Supplementary material for: Prospective observational study and serosurvey of SARS-CoV-2 infection in asymptomatic healthcare workers at a Canadian tertiary care center
Source: PLoS One. 2021 Feb 16;16(2):e0247258. doi: 10.1371/journal.pone.0247258 (PMC7886177; doi:10.1371/journal.pone.0247258)

S1 FIG: Sequencing of SARS-CoV-2 from 6/9 healthcare workers with active infection (numbers on y-axis correspond to those in Supplementary Table 3). A multiple sequence alignment of all consensus reads and the MN908947.3 reference was generated, then used to build a phylogenetic tree using augur (https://github.com/nextstrain/augur). Variants were called using scripts developed as part of the nCoV-tools package (https://github.com/jts/ncov-tools). Sites with single base substitutions are shown, with N indicating no coverage at the site. For genome completeness, a cut-off of 75% was used to sequence samples. Three of the nine samples did not meet this cut-off. Genome completeness ranged between 83.7-97.1%. Results demonstrate 3 variants. The D614G mutation, which occurs at nucleotide position 23403 of the reference strain, is indicated with an arrow.

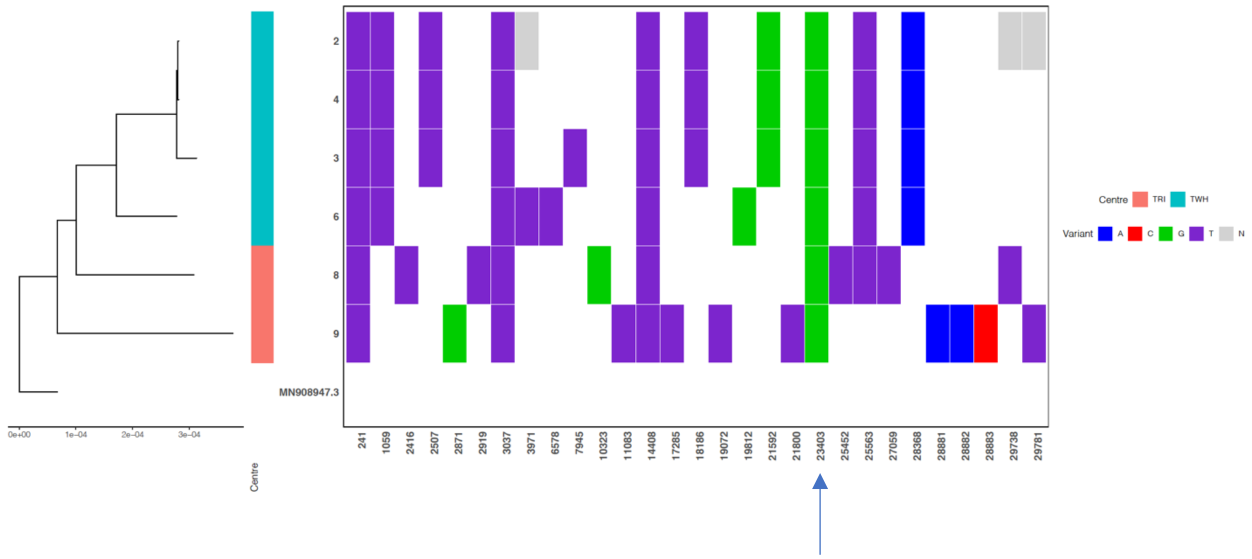

Supplement: S1 Fig — Numbers on y-axis correspond to those in S3 Table. A multiple sequence alignment of all consensus reads and the MN908947.3 reference was generated, then used to build a phylogenetic tree using augur (https://github.com/nextstrain/augur). Variants were called using scripts developed as part of the nCoV-tools package (https://github.com/jts/ncov-tools). Sites with single base substitutions are shown, with N indicating no coverage at the site. For genome completeness, a cut-off of 75% was used to sequence samples. Three of the nine samples did not meet this cut-off. Genome completeness ranged between 83.7–97.1%. Results demonstrate 3 variants. The D614G mutation, which occurs at nucleotide position 23403 of the reference strain, is indicated with an arrow. (PDF) [file pone.0247258.s001.pdf]
