# Supplementary material for: Prospective observational study and serosurvey of SARS-CoV-2 infection in asymptomatic healthcare workers at a Canadian tertiary care center
Source: PLoS One. 2021 Feb 16;16(2):e0247258. doi: 10.1371/journal.pone.0247258 (PMC7886177; doi:10.1371/journal.pone.0247258)
Supplement: S2 Fig — A) Images of 2-color arrays probed with secondary antibodies only, pre-COVID serum (negative control) and COVID+ serum (positive control). Antigens were spotted in triplicate; green indicates IgG reactivity, whereas red indicates IgM reactivity. On the array probed only with secondary antibodies, only human IgG and human IgM are detected. On the array probed with pre-COVID serum, reactivity against common community coronavirus antigens is detected. On the array probed with COVID+ serum, there are additional SARS-CoV-2 reactivities detected (boxes). Array features are approximately 500 μm in diameter. B) and C) Linearity studies using serial dilutions of COVID+ serum. Graph B shows MFI-B plotted against serum dilutions, whereas Graph C shows log2 transformed MFI-B. Linear responses are observed over a wide range of serum dilutions using log2 transformed MFI-B. Antibody responses become non-linear as MFI-B approaches saturation levels (MFI-B > 60,000). Abbreviations: MFI-B—median fluorescent intensity minus background. (PDF) [file pone.0247258.s002.pdf]

S2 FIG: Images of antigen microarrays and determination of linearity of the array assay. A) Images of 2-color arrays probed with secondary antibodies only, pre-COVID serum (negative control) and COVID+ serum (positive control). Antigens were spotted in triplicate; green indicates IgG reactivity, whereas red indicates IgM reactivity. On the array probed only with secondary antibodies, only human IgG and human IgM are detected. On the array probed with pre-COVID serum, reactivity against common community coronavirus antigens is detected. On the array probed with COVID+ serum, there are additional SARS-CoV-2 reactivities detected (boxes). Array features are approximately 500 μm in diameter. B) and C) Linearity studies using serial dilutions of COVID+ serum. Graph B shows MFI-B plotted against serum dilutions, whereas Graph C shows $\log_2$ transformed MFI-B. Linear responses are observed over a wide range of serum dilutions using $\log_2$ transformed MFI-B. Antibody responses become non-linear as MFI-B approaches saturation levels (MFI-B > 60,000). MFI-B, median fluorescent intensity minus background.

A

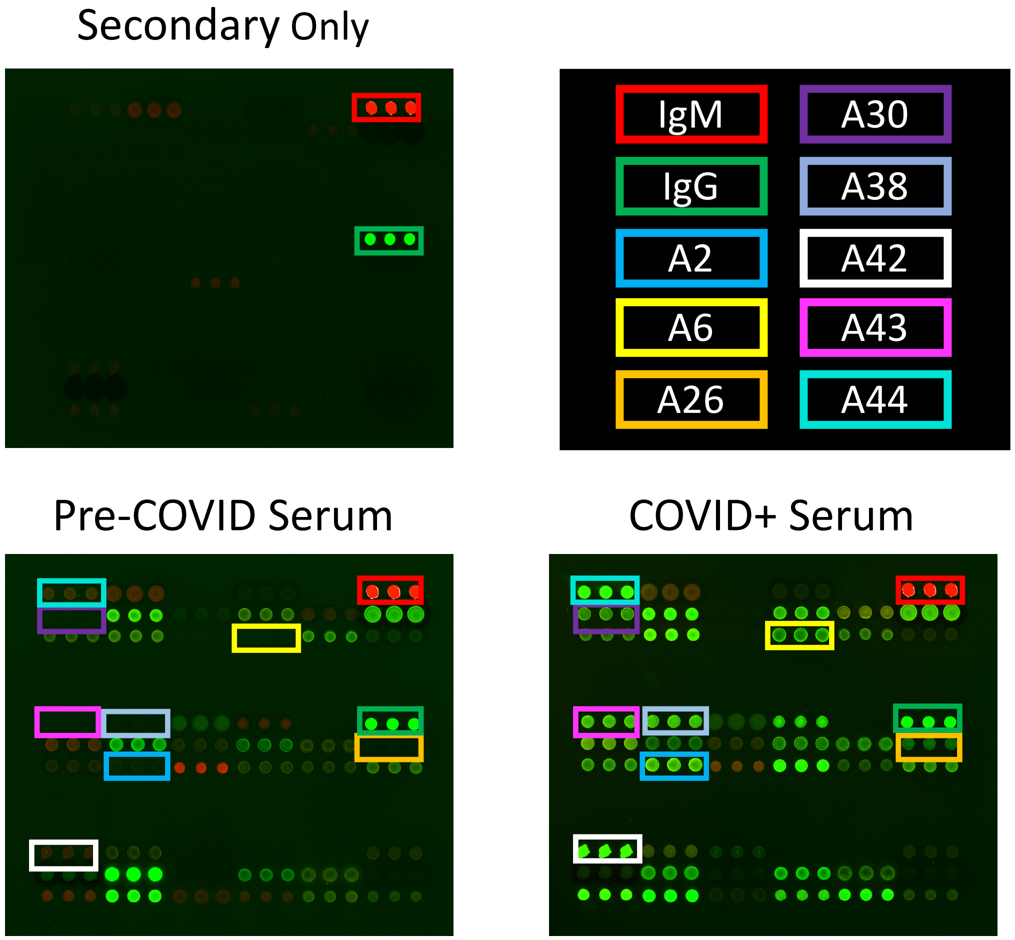

Secondary Only
IgM
A30
IgG
A38
A2
A42
A6
A43
A26
A44
Pre-COVID Serum
COVID+ Serum

B

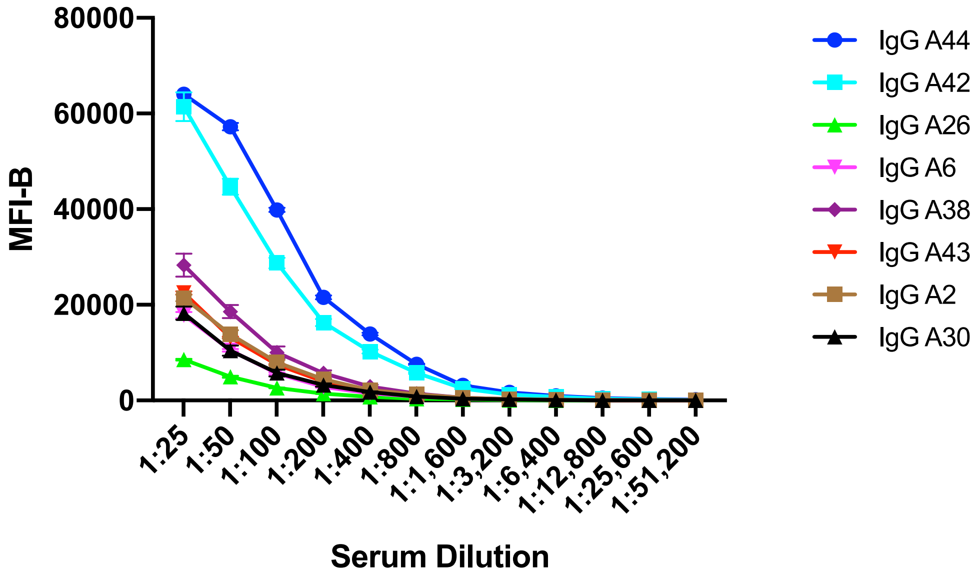

80000
60000
40000
20000
0
MFI-B
1:25
1:50
1:100
1:200
1:400
1:800
1:1,600
1:3,200
1:6,400
1:12,800
1:25,600
1:51,200
Serum Dilution
IgG A44
IgG A42
IgG A26
IgG A6
IgG A38
IgG A43
IgG A2
IgG A30

C

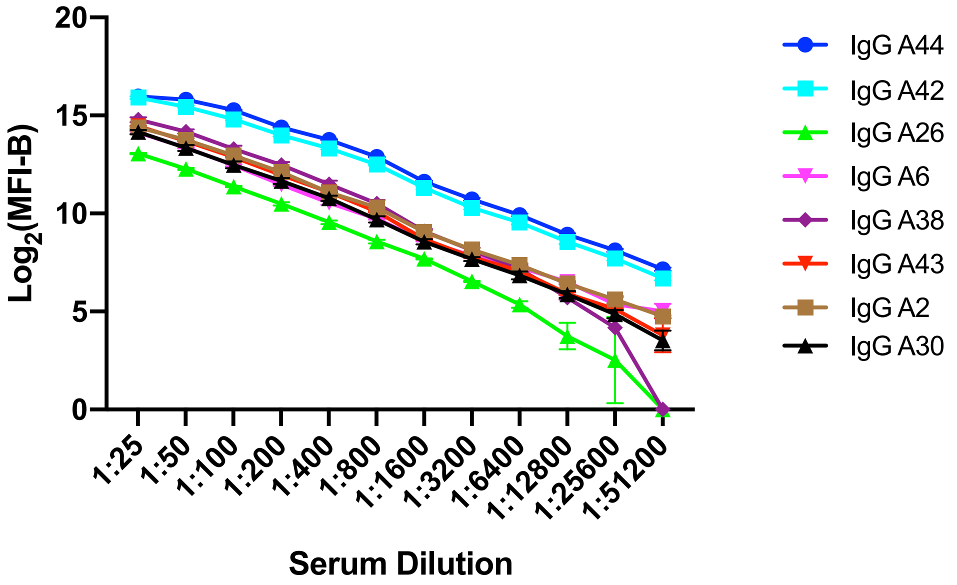

20
15
10
5
0
Log2(MFI-B)
1:25
1:50
1:100
1:200
1:400
1:800
1:1600
1:3200
1:6400
1:12800
1:25600
1:51200
Serum Dilution
IgG A44
IgG A42
IgG A26
IgG A6
IgG A38
IgG A43
IgG A2
IgG A30
