# Supplementary material for: Prospective observational study and serosurvey of SARS-CoV-2 infection in asymptomatic healthcare workers at a Canadian tertiary care center
Source: PLoS One. 2021 Feb 16;16(2):e0247258. doi: 10.1371/journal.pone.0247258 (PMC7886177; doi:10.1371/journal.pone.0247258)

S3 FIG: Heatmap of the 39 antigen reactivities upregulated in COVID+ patients as determined by significance analysis of microarrays. The COVID+ samples (n=7) form a separate cluster from the pre-COVID samples (n=18) using a hierarchical clustering algorithm. Yellow indicates high reactivity, whereas blue indicates low reactivity.

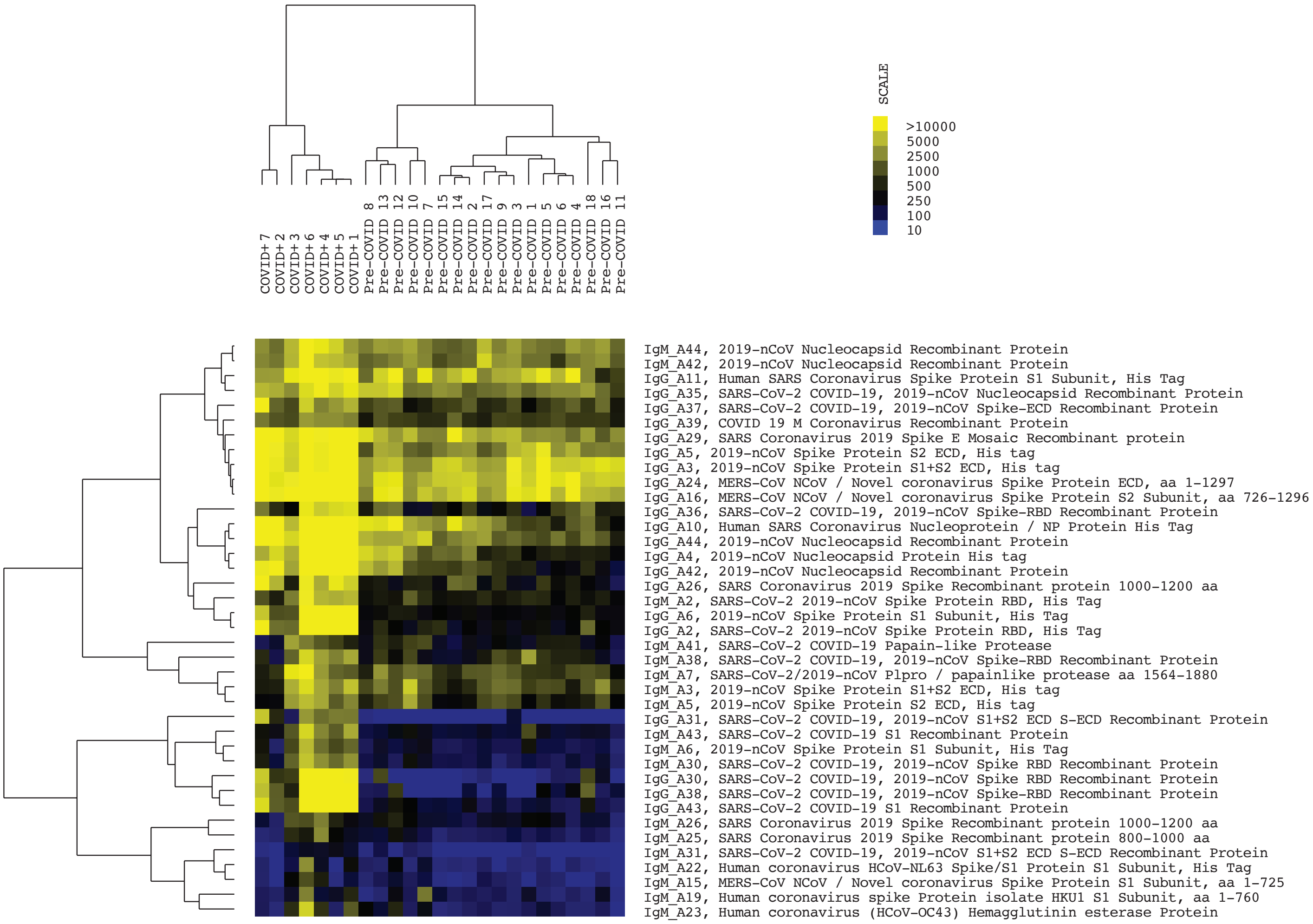

Supplement: S3 Fig — The COVID+ samples (n = 7) form a separate cluster from the pre-COVID samples (n = 18) using a hierarchical clustering algorithm. Yellow indicates high reactivity, whereas blue indicates low reactivity. (PDF) [file pone.0247258.s003.pdf]
