# Supplementary material for: Prospective observational study and serosurvey of SARS-CoV-2 infection in asymptomatic healthcare workers at a Canadian tertiary care center
Source: PLoS One. 2021 Feb 16;16(2):e0247258. doi: 10.1371/journal.pone.0247258 (PMC7886177; doi:10.1371/journal.pone.0247258)
Supplement: S1 Table — (DOCX) [file pone.0247258.s004.docx]

**S1 Table: Viral Antigens included in Protein Microarray.**

| Antigen # | Antigen | Virus | Source | Catalog # | Expression System |
| --- | --- | --- | --- | --- | --- |
| A1 | Human coronavirus (HCoV-229E) Spike Protein (S1 Subunit, His Tag) | Community Coronavirus | Sino Biological | 40601-V08H | HEK293 |
| A2 | SARS-CoV-2 (2019-nCoV) Spike Protein (RBD, His Tag) | SARS-CoV-2 | Sino Biological | 40592-V08H | HEK293 |
| A3 | 2019-nCoV Spike Protein (S1+S2 ECD, His tag) | SARS-CoV-2 | Sino Biological | 40589-V08B1 | Baculovirus-Insect Cells |
| A4 | 2019-nCoV Nucleocapsid Protein (His tag) | SARS-CoV-2 | Sino Biological | 40588-V08B | Baculovirus-Insect Cells |
| A5 | 2019-nCoV Spike Protein (S2 ECD, His tag) | SARS-CoV-2 | Sino Biological | 40590-V08B | Baculovirus-Insect Cells |
| A6 | 2019-nCoV Spike Protein (S1 Subunit, His Tag) | SARS-CoV-2 | Sino Biological | 40591-V08H | HEK293 |
| A7 | SARS-CoV-2/2019-nCoV Plpro / papainlike protease (aa 1564-1880, His Tag) | SARS-CoV-2 | Sino Biological | 40593-V07E | E. Coli |
| A8 | 2019-nCoV Spike Protein (S1 Subunit, His tag) | SARS-CoV-2 | Sino Biological | 40591-V08B1 | Baculovirus-Insect Cells |
| A9 | SARS-CoV-2 (2019-nCoV) Methyltransferase / ME-his Recombinant Protein | SARS-CoV-2 | Sino Biological | 40598-V07E | E. Coli |
| A10 | Human SARS Coronavirus Nucleoprotein / NP Protein (His Tag) | SARS-CoV | Sino Biological | 40143-V08B | Baculovirus-Insect Cells |
| A11 | Human SARS Coronavirus Spike Protein (S1 Subunit, His Tag) | SARS-CoV | Sino Biological | 40150-V08B1 | Baculovirus-Insect Cells |
| A12 | Human SARS Coronavirus Spike Protein (RBD, His Tag) | SARS-CoV | Sino Biological | 40150-V08B2 | Baculovirus-Insect Cells |
| A13 | SARS-CoV (strain WH20) Plpro / papain-like protease (His Tag) | SARS-CoV | Sino Biological | 40524-V08E | E. Coli |
| A14 | MERS-CoV (NCoV / Novel coronavirus) Nucleoprotein / NP protein (His Tag) | MERS-CoV | Sino Biological | 40068-V08B | Baculovirus-Insect Cells |
| A15 | MERS-CoV (NCoV / Novel coronavirus) Spike Protein (S1 Subunit, aa 1-725, His Tag) | MERS-CoV | Sino Biological | 40069-V08H | HEK293 |
| A16 | MERS-CoV (NCoV / Novel coronavirus) Spike Protein (S2 Subunit, aa 726-1296, His Tag) | MERS-CoV | Sino Biological | 40070-V08B | Baculovirus-Insect Cells |
| A17 | MERS-CoV (NCoV / Novel coronavirus) Spike Protein fragment (RBD, aa 367-606, His Tag) | MERS-CoV | Sino Biological | 40071-V08B1 | Baculovirus-Insect Cells |
| A18 | MERS-CoV (NCoV / Novel coronavirus) Spike Protein (S1 Subunit, aa 1-725, His Tag) | MERS-CoV | Sino Biological | 40069-V08B1 | Baculovirus-Insect Cells |
| A19 | Human coronavirus spike glycoprotein Protein (isolate HKU1) (S1 Subunit, aa 1-760, His Tag) | Community Coronavirus | Sino Biological | 40021-V08H | HEK293 |
| A20 | Human coronavirus HKU1 (isolate N5) (HCoV-HKU1) Spike/S1 Protein (S1 Subunit, His Tag) | Community Coronavirus | Sino Biological | 40602-V08H | HEK293 |
| A21 | Human coronavirus (HCoV-229E) Spike Protein (S1+S2 ECD, His Tag) | Community Coronavirus | Sino Biological | 40605-V08B | Baculovirus-Insect Cells |
| A22 | Human coronavirus (HCoV-NL63) Spike/S1 Protein (S1 Subunit, His Tag) | Community Coronavirus | Sino Biological | 40600-V08H | HEK293 |
| A23 | Human coronavirus (HCoV-OC43) Hemagglutinin esterase Protein (His Tag) | Community Coronavirus | Sino Biological | 40603-V08H | HEK293 |
| A24 | MERS-CoV (NCoV / Novel coronavirus) Spike Protein (ECD, aa 1-1297, His Tag) | MERS-CoV | Sino Biological | 40069-V08B | Baculovirus-Insect Cells |
| A25 | SARS Coronavirus 2019 Spike Recombinant protein (800-1000 aa) | SARS-CoV-2 | ProSci Inc | 39-125 | E. Coli |
| A26 | SARS Coronavirus 2019 Spike Recombinant protein (1000-1200 aa) | SARS-CoV-2 | ProSci Inc | 39-126 | E. Coli |
| A27 | SARS Coronavirus 2019 Nucleocapsid Recombinant protein | SARS-CoV-2 | ProSci Inc | 39-113 | E. Coli |
| A28 | SARS Coronavirus 2019 Nucleocapsid Mosaic Recombinant protein | SARS-CoV-2 | ProSci Inc | 39-115 | E. Coli |
| A29 | SARS Coronavirus 2019 Spike E Mosaic Recombinant protein | SARS-CoV-2 | ProSci Inc | 39-114 | E. Coli |
| A30 | SARS-CoV-2 (COVID-19, 2019-nCoV) Spike RBD Recombinant Protein | SARS-CoV-2 | ProSci Inc | 10-100 | Human cells |
| A31 | SARS-CoV-2 (COVID-19, 2019-nCoV) S1+S2 ECD (S-ECD) Recombinant Protein | SARS-CoV-2 | ProSci Inc | 10-108 | Human cells |
| A32 | SARS-CoV-2 (COVID-19, 2019-nCoV) ORF8 Recombinant Protein | SARS-CoV-2 | ProSci Inc | 10-002 | E. Coli |
| A33 | SARS-CoV-2 (COVID-19, 2019-nCoV) ORF3a Recombinant Protein | SARS-CoV-2 | ProSci Inc | 10-005 | E. Coli |
| A34 | SARS-CoV-2 (COVID-19, 2019-nCoV) Spike Recombinant Protein | SARS-CoV-2 | ProSci Inc | 10-006 | E. Coli |
| A35 | SARS-CoV-2 (COVID-19, 2019-nCoV) Nucleocapsid Recombinant Protein | SARS-CoV-2 | ProSci Inc | 10-007 | E. Coli |
| A36 | SARS-CoV-2 (COVID-19, 2019-nCoV) Spike-RBD Recombinant Protein | SARS-CoV-2 | ProSci Inc | 10-008 | Sf21 cells |
| A37 | SARS-CoV-2 (COVID-19, 2019-nCoV) Spike-ECD Recombinant Protein | SARS-CoV-2 | ProSci Inc | 10-011 | Sf21 cells |
| A38 | SARS-CoV-2 (COVID-19, 2019-nCoV) Spike-RBD Recombinant Protein | SARS-CoV-2 | ProSci Inc | 10-015 | Human cells |
| A39 | COVID 19 M Coronavirus Recombinant Protein | SARS-CoV-2 | mybiosource.com | MBS8574735 | E. Coli |
| A40 | SARS-CoV-2 (COVID-19) 3C-like Proteinase | SARS-CoV-2 | ProSci Inc | 10-116 | E. Coli |
| A41 | SARS-CoV-2 (COVID-19) Papain-like Protease | SARS-CoV-2 | ProSci Inc | 10-119 | E. Coli |
| A42 | 2019-nCoV Nucleocapsid Recombinant Protein | SARS-CoV-2 | ProSci Inc | 97-085 | HEK293 |
| A43 | SARS-CoV-2 (COVID-19) S1 Recombinant Protein | SARS-CoV-2 | ProSci Inc | 97-087 | HEK293 |
| A44 | 2019-nCoV Nucleocapsid Recombinant Protein | SARS-CoV-2 | ProSci Inc | 97-077 | E. Coli |
| A45 | 2019-nCoV Envelope Recombinant Protein | SARS-CoV-2 | ProSci Inc | 97-082 | E. Coli |
