# Supplementary material for: Prospective observational study and serosurvey of SARS-CoV-2 infection in asymptomatic healthcare workers at a Canadian tertiary care center
Source: PLoS One. 2021 Feb 16;16(2):e0247258. doi: 10.1371/journal.pone.0247258 (PMC7886177; doi:10.1371/journal.pone.0247258)
Supplement: S2 Table — (DOCX) [file pone.0247258.s005.docx]

**S2 Table: Characteristics of health care workers undergoing nasopharyngeal swab (in Cohort 1) and serology testing.**

| **Variable** | **Nasopharyngeal SARS-CoV-2 PCR**  **N=1669** | **Serology**  **N=996** |
| --- | --- | --- |
| Age (years); mean ± s.d. | 40.3 ± 11.3 | 40.8 ± 11.1 |
| Sex (M/F/Other) | 356/1312/1 | 215/781 |
| Occupation  Nurse  Physician  Allied Health  Other  Not specified | 655 (39.2%)  152 (9.1%)  446 (26.7%)  396 (23.7%)  20 (1.2%) | 361(36.2%)  101 (10.1%)  261 (26.2%)  272 (27.3%)  1 (0.1%) |
| Lives with children <12 years of age | 452/1555 (29.1%) | 266/927 (28.7%) |
| Directly looked after COVID patient in the last 2 weeks | 472/1555 (30.4%) | 255/927 (27.5%) |
| Travel in the last 2 weeks | 2/1555 (0.13%) | 2/927 (0.22%) |
