# Supplementary material for: Prospective observational study and serosurvey of SARS-CoV-2 infection in asymptomatic healthcare workers at a Canadian tertiary care center
Source: PLoS One. 2021 Feb 16;16(2):e0247258. doi: 10.1371/journal.pone.0247258 (PMC7886177; doi:10.1371/journal.pone.0247258)
Supplement: S3 Table — (DOCX) [file pone.0247258.s006.docx]

**S3 Table: Asymptomatic Healthcare workers that had positive SARS-CoV-2 PCR (n=9) in Cohort 1.**

| **Study Code** | **Age/Sex** | **Occupation** | **Direct Care of COVID patient** | **Development of Symptoms in the 2 weeks following testing** | **Convalescent serology * (anti-NP index)**  **Days post-PCR+** |
| --- | --- | --- | --- | --- | --- |
| 1 | 27/F | Nurse | No | No | Not done |
| 2 | 44/F | Nurse | Yes | Yes | 1.4 (Positive) |
| 3 | 52/M | Nurse | Yes | Yes | Not done |
| 4 | 26/F | Nurse | No | Yes | 7.6 (Positive) |
| 5 | 26/F | Nurse | No | Yes | 5.8 (Positive) |
| 6 | 37/F | Nurse | No | No | 6.7 (Positive) |
| 7 | 32/F | Allied Health | No | No | 0.03 (Negative) |
| 8 | 40/F | Nurse | No | No | Not done |
| 9 | 33/F | Nurse | Yes | No | 0.1 (Negative) |

*serum taken 2-8 weeks after positive nasopharyngeal swab
