# Supplementary material for: Prospective observational study and serosurvey of SARS-CoV-2 infection in asymptomatic healthcare workers at a Canadian tertiary care center
Source: PLoS One. 2021 Feb 16;16(2):e0247258. doi: 10.1371/journal.pone.0247258 (PMC7886177; doi:10.1371/journal.pone.0247258)
Supplement: S4 Table — (DOCX) [file pone.0247258.s007.docx]

**S4 Table: Healthcare Workers that were SARS-CoV-2 anti-nucleoprotein (NP) IgG positive (n=14).**

| **Study Number** | **Age/Sex** | **Occupation** | **Direct Care of COVID patient** | **History of Recent Compatible Symptoms** | **SARS-CoV-2 PCR done on same day** |
| --- | --- | --- | --- | --- | --- |
| 1* | 27/F | Nurse | - | - | - |
| 2* | 45/F | Allied Health | + | - | - |
| 3 | 71/M | Physician | - | + | ND |
| 4 | 38/M | Nurse | - | - | - |
| 5 | 44/M | Nurse | - | + | - |
| 6 | 55/F | Nurse | - | - | - |
| 7 | 40/F | Allied Health | - | - | - |
| 8 | 61/F | Nurse | - | - | - |
| 9 | 46/F | Nurse | - | - | - |
| 10 | 39/F | Nurse | - | + | - |
| 11 | 36/F | Allied Health | - | - | - |
| 12 | 46/F | Allied Health | - | - | - |
| 13 | 58/F | Administrator | - | - | - |
| 14 | 36/M | Allied Health | - | + | - |

ND, not done

*also anti-Spike IgG positive
