# Supplementary material for: Prospective observational study and serosurvey of SARS-CoV-2 infection in asymptomatic healthcare workers at a Canadian tertiary care center
Source: PLoS One. 2021 Feb 16;16(2):e0247258. doi: 10.1371/journal.pone.0247258 (PMC7886177; doi:10.1371/journal.pone.0247258)
Supplement: S5 Table — (DOCX) [file pone.0247258.s008.docx]

**S5 Table: List of antigen reactivities upregulated in COVID+ patients as determined by significance analysis of microarrays (fold change > 2, false discovery rate < 1%).**

| **Antigen** | **Score** | **Fold Change** | **q-value(%)** |
| --- | --- | --- | --- |
| IgG_A43, SARS-CoV-2 (COVID-19) S1 Recombinant Protein | 7.75 | 71.38 | 0 |
| IgG_A31, SARS-CoV-2 (COVID-19, 2019-nCoV) S1+S2 ECD (S-ECD) Recombinant Protein | 7.36 | 36.60 | 0 |
| IgG_A2, SARS-CoV-2 (2019-nCoV) Spike Protein (RBD, His Tag) | 6.92 | 44.40 | 0 |
| IgG_A30, SARS-CoV-2 (COVID-19, 2019-nCoV) Spike RBD Recombinant Protein | 6.91 | 113.45 | 0 |
| IgG_A6, 2019-nCoV Spike Protein (S1 Subunit, His Tag) | 6.66 | 26.43 | 0 |
| IgG_A44, 2019-nCoV Nucleocapsid Recombinant Protein | 6.50 | 29.81 | 0 |
| IgG_A38, SARS-CoV-2 (COVID-19, 2019-nCoV) Spike-RBD Recombinant Protein | 6.12 | 79.78 | 0 |
| IgG_A42, 2019-nCoV Nucleocapsid Recombinant Protein | 5.94 | 43.87 | 0 |
| IgM_A30, SARS-CoV-2 (COVID-19, 2019-nCoV) Spike RBD Recombinant Protein | 5.18 | 12.15 | 0 |
| IgG_A26, SARS Coronavirus 2019 Spike Recombinant protein (1000-1200 aa) | 5.05 | 17.16 | 0 |
| IgG_A5, 2019-nCoV Spike Protein (S2 ECD, His tag) | 4.97 | 9.98 | 0 |
| IgG_A10, Human SARS Coronavirus Nucleoprotein / NP Protein (His Tag) | 4.53 | 17.43 | 0 |
| IgG_A29, SARS Coronavirus 2019 Spike E Mosaic Recombinant protein | 4.47 | 8.03 | 0 |
| IgG_A4, 2019-nCoV Nucleocapsid Protein (His tag) | 4.31 | 13.08 | 0 |
| IgM_A2, SARS-CoV-2 (2019-nCoV) Spike Protein (RBD, His Tag) | 4.29 | 6.03 | 0 |
| IgM_A43, SARS-CoV-2 (COVID-19) S1 Recombinant Protein | 4.25 | 8.07 | 0 |
| IgM_A6, 2019-nCoV Spike Protein (S1 Subunit, His Tag) | 3.99 | 7.44 | 0 |
| IgG_A16, MERS-CoV (NCoV / Novel coronavirus) Spike Protein (S2 Subunit, aa 726-1296, His Tag) | 3.81 | 5.54 | 0 |
| IgG_A37, SARS-CoV-2 (COVID-19, 2019-nCoV) Spike-ECD Recombinant Protein | 3.51 | 4.36 | 0 |
| IgG_A39, COVID 19 M Coronavirus Recombinant Protein | 3.27 | 2.62 | 0 |
| IgG_A3, 2019-nCoV Spike Protein (S1+S2 ECD, His tag) | 3.25 | 3.89 | 0 |
| IgG_A36, SARS-CoV-2 (COVID-19, 2019-nCoV) Spike-RBD Recombinant Protein | 2.99 | 6.01 | 0 |
| IgM_A31, SARS-CoV-2 (COVID-19, 2019-nCoV) S1+S2 ECD (S-ECD) Recombinant Protein | 2.92 | 2.82 | 0 |
| IgG_A24, MERS-CoV (NCoV / Novel coronavirus) Spike Protein (ECD, aa 1-1297, His Tag) | 2.90 | 3.24 | 0 |
| IgG_A35, SARS-CoV-2 (COVID-19, 2019-nCoV) Nucleocapsid Recombinant Protein | 2.57 | 2.78 | 0 |
| IgM_A42, 2019-nCoV Nucleocapsid Recombinant Protein | 2.16 | 2.41 | 0 |
| IgM_A23, Human coronavirus (HCoV-OC43) Hemagglutinin esterase Protein (His Tag) | 2.15 | 4.07 | 0 |
| IgM_A38, SARS-CoV-2 (COVID-19, 2019-nCoV) Spike-RBD Recombinant Protein | 2.14 | 3.78 | 0 |
| IgM_A25, SARS Coronavirus 2019 Spike Recombinant protein (800-1000 aa) | 2.12 | 2.98 | 0 |
| IgM_A15, MERS-CoV (NCoV / Novel coronavirus) Spike Protein (S1 Subunit, aa 1-725, His Tag) | 1.99 | 2.67 | 0 |
| IgM_A44, 2019-nCoV Nucleocapsid Recombinant Protein | 1.99 | 2.11 | 0 |
| IgM_A26, SARS Coronavirus 2019 Spike Recombinant protein (1000-1200 aa) | 1.75 | 2.43 | 0 |
| IgM_A22, Human coronavirus (HCoV-NL63) Spike/S1 Protein (S1 Subunit, His Tag) | 1.75 | 2.64 | 0 |
| IgM_A7, SARS-CoV-2/2019-nCoV Plpro / papainlike protease (aa 1564-1880, His Tag) | 1.71 | 2.34 | 0 |
| IgM_A5, 2019-nCoV Spike Protein (S2 ECD, His tag) | 1.61 | 2.34 | 0 |
| IgG_A11, Human SARS Coronavirus Spike Protein (S1 Subunit, His Tag) | 1.58 | 2.47 | 0 |
| IgM_A19, Human coronavirus spike glycoprotein Protein (isolate HKU1) (S1 Subunit, aa 1-760, His Tag) | 1.48 | 2.41 | 0 |
| IgM_A3, 2019-nCoV Spike Protein (S1+S2 ECD, His tag) | 1.47 | 2.12 | 0 |
| IgM_A41, SARS-CoV-2 (COVID-19) Papain-like Protease | 1.46 | 2.20 | 0 |
